# Liquid chromatography/mass spectrometry analysis of exhaled leukotriene B_4 _in asthmatic children

**DOI:** 10.1186/1465-9921-6-119

**Published:** 2005-10-19

**Authors:** Paolo Montuschi, Simona Martello, Marialinda Felli, Chiara Mondino, Peter J Barnes, Marcello Chiarotti

**Affiliations:** 1 Department of Pharmacology, Faculty of Medicine, Catholic University of the Sacred Heart, Rome, Italy; 2 Department of Forensic Medicine, Faculty of Medicine, Catholic University of the Sacred Heart, Rome, Italy; 3 Department of Immunodermatology, Istituto Dermopatico dell'Immacolata, IDI, IRCCS, Rome, Italy; 4 Department of Thoracic Medicine, Imperial College, School of Medicine at the National Heart and Lung Institute, London, UK

## Abstract

**Background:**

The role of leukotriene (LT) B_4_, a potent inflammatory mediator, in atopic asthmatic and atopic nonasthmatic children is largely unknown. The lack of a gold standard technique for measuring LTB_4 _in exhaled breath condensate (EBC) has hampered its quantitative assessment in this biological fluid. We sought to measure LTB_4 _in EBC in atopic asthmatic children and atopic nonasthmatic children. Exhaled nitric oxide (NO) was measured as an independent marker of airway inflammation.

**Methods:**

Fifteen healthy children, 20 atopic nonasthmatic children, 25 steroid-naïve atopic asthmatic children, and 22 atopic asthmatic children receiving inhaled corticosteroids were studied. The study design was of cross-sectional type. Exhaled LTB_4 _concentrations were measured using liquid chromatography/mass spectrometry-mass spectrometry (LC/MS/MS) with a triple quadrupole mass spectrometer. Exhaled NO was measured by chemiluminescence with a single breath on-line method. LTB_4 _values were expressed as the total amount (in pg) of eicosanoid expired in the 15-minute breath test. Kruskal-Wallis test was used to compare groups.

**Results:**

Compared with healthy children [87.5 (82.5–102.5) pg, median and interquartile range], exhaled LTB_4 _was increased in steroid-naïve atopic asthmatic [255.1 (175.0–314.7) pg, p < 0.001], but not in atopic nonasthmatic children [96.5 (87.3–102.5) pg, p = 0.59)]. Asthmatic children who were receiving inhaled corticosteroids had lower concentrations of exhaled LTB_4 _than steroid-naïve asthmatics [125.0 (25.0–245.0) pg vs 255.1 (175.0–314.7) pg, p < 0.01, respectively]. Exhaled NO was higher in atopic nonasthmatic children [16.2 (13.5–22.4) ppb, p < 0.05] and, to a greater extent, in atopic steroid-naïve asthmatic children [37.0 (31.7–57.6) ppb, p < 0.001] than in healthy children [8.3 (6.1–9.9) ppb]. Compared with steroid-naïve asthmatic children, exhaled NO levels were reduced in asthmatic children who were receiving inhaled corticosteroids [15.9 (11.5–31.7) ppb, p < 0.01].

**Conclusion:**

In contrast to exhaled NO concentrations, exhaled LTB_4 _values are selectively elevated in steroid-naïve atopic asthmatic children, but not in atopic nonasthmatic children. Although placebo control studies are warranted, inhaled corticosteroids seem to reduce exhaled LTB_4 _in asthmatic children. LC/MS/MS analysis of exhaled LTB_4 _might provide a non-invasive, sensitive, and quantitative method for airway inflammation assessment in asthmatic children.

## Background

Chronic airway inflammation, the primary pathophysiological feature of asthma, underlies asthma symptoms and might have a role in airway and lung remodelling [1]. Elevated airway inflammation often precedes the onset of symptoms or airway limitation [1]. Quantification of airway inflammation is difficult as it requires invasive techniques or measurement of biomarkers in plasma or urine which reflect systemic rather than lung inflammation. Bronchoscopy with bronchoalveolar lavage remains the gold standard to assess airway inflammation, but invasiveness makes it unethical as a routine method particularly in children [2]. Sputum induction is less invasive [3], although this technique is particularly difficult to apply in young children and itself induces airway inflammation [4].

This has led to a search for non-invasive ways to measure airway and lung inflammation in order to aid diagnosis, to assess response to anti-inflammatory treatments, to predict loss of disease control and to assess the response to novel treatments [5]. There is increasing evidence that measurement of biomarkers in the breath may reflect pulmonary disease [6]. Direct sampling form the lung has major advantages compared with sampling form the blood or urine, when dilution and metabolism of inflammatory markers arising in the lungs makes interpretation very difficult. Exhaled nitric oxide (NO) measurement is currently the only completely noninvasive method for quantifying airway inflammation in patients with asthma who are not treated with corticosteroids [2,7]. However, as several inflammatory mediators are involved in asthma, identification of other exhaled markers might contribute to a better understanding of its complex pathophysiology. Exhaled breath condensate (EBC) is a completely noninvasive method for collecting airway secretions [8,9]. Cysteynyl-leukotrienes play an important role in the pathophysiology of asthma and their regulation is relevant for the management of this disease [10]. However, the role of leukotriene (LT) B_4_, a potent chemoattractant for neutrophils, in asthma is largely unknown. Using immunoassays, LTB_4 _has been detected in EBC in healthy subjects and found increased in asthmatic adults [11,12] and children [13,14]. However, the presence of LTB_4 _in EBC has not been confirmed by more specific analytical techniques. Moreover, a large variation in exhaled LTB_4 _concentrations in patients with similar clinical and functional features has been reported in different studies [13-15]. This may be partially explained by the lack of validation of commercially available immunoassay kits which were used to measure exhaled LTB_4 _in these studies [16]. Mass spectrometry, the reference analytical technique, is required for an accurate quantitative assessment of exhaled LTB_4_. In the present study, we aimed to measure LTB_4 _concentrations in EBC with liquid chromatography/mass spectrometry-mass spectrometry (LC/MS/MS) using deuterated (d_4_) LTB_4 _as internal standard in healthy children, atopic nonasthmatic children, steroid-naïve atopic asthmatic children, and atopic asthmatic children who were treated with inhaled corticosteroids. We also measured exhaled NO as an independent marker of airway inflammation.

## Methods

### Study subjects

Four groups of children were studied: 15 healthy children, 20 atopic nonasthmatic children, 25 steroid-naïve atopic asthmatic children, and 22 atopic asthmatic children who were receiving inhaled corticosteroids (Table 1). Atopic nonasthmatic and atopic asthmatic children were recruited from the Asthma and Allergy outpatient clinic, Istituto Dermopatico dell'Immacolata, Rome, Italy. The diagnosis and classification of asthma was based on clinical history and examination and pulmonary function parameters according to the Guidelines for the Diagnosis and Management of Asthma issued by the National Heart, Lung, and Blood Institute of the National Institutes of Health [17]. Steroid-naïve atopic asthmatic children had mild intermittent asthma with symptoms less often than twice a week, forced expiratory volume in one second (FEV_1_) of 80% or greater of predicted value and reversibility of 12% or greater to salbutamol, or positive provocation test result with methacholine or exercise. They were not taking any regular medication but used inhaled short-acting β_2_-agonists as needed for symptom relief. They were excluded from the study if they had used inhaled corticosteroids or LT receptor antagonists in the previous 4 weeks. Atopy was confirmed by skin prick testing. Steroid-treated atopic asthmatic children had persistent mild-to-moderate asthma and were receiving maintenance therapy with either 100 μg/day (10 children) or 200 μg/day (12 children) of inhaled fluticasone propionate administered by means of diskus (GSK, Uxbridge, United Kingdom) at a constant dose for at least 8 weeks. They had not been taking LT receptor antagonists for at least one month. Atopy was confirmed by skin prick tests.

**Table 1:** Subject characteristics^§^

	Healthy children	Atopic nonasthmatic children	Steroid-naïve atopic asthmatic children	Steroid-treated atopic asthmatic children
n	15	20	25	22
Sex, F/M	8/7	9/11	12/13	10/12
Age, yr	10 ± 1	9 ± 1	10 ± 1	10 ± 1
FEV_1_, % pred	101.5 ± 1.8	100.8 ± 2.3	96.1 ± 1.4	97.5 ± 2.3
FVC, % pred	102.7 ± 2.8	103.2 ± 2.5	100.4 ± 2.3	101.5 ± 2.7
FEV_1_/FVC, %	99.8 ± 1.5	97.5 ± 2.6	87.1 ± 2.3*	85.8 ± 2.5*
FEF_25–75%_	104.0 ± 3.8	101.8 ± 4.1	68.2 ± 3.9**	83.7 ± 4.5*

^§ ^Data are expressed as numbers or mean ± SEM.

* P < 0.05 and ** P < 0.01 compared with healthy children. Steroid-treated asthmatic children were receiving either 100 μg/day or 200 μg/day of inhaled fluticasone at a constant dose for at least 8 weeks.

Atopic nonasthmatic children had a clinical history of atopy and positive skin test results. All had allergic rhinitis and were either sensitized to perennial allergens or were recently exposed to a relevant allergen.

Healthy control children had no history of asthma and atopic disease and negative skin prick test results. They were recruited from children of staff.

Study group children had no upper respiratory tract infections in the previous 3 weeks. Children were excluded from the study if they had used oral corticosteroids in the previous 4 weeks or non-steroidal anti-inflammatory drugs in the last 2 weeks.

### Study design

The study was of cross-sectional design. Children attended the Department of Pharmacology, Catholic University of the Sacred Heart, Rome, Italy, for clinical examination, EBC collection, spirometry and skin prick testing. Informed consent was obtained by parents and the study was approved by the Ethics Committee of the Catholic University of the Sacred Heart, Rome, Italy.

Day-to-day repeatability for LTB_4 _measurements was assessed in 20 children with asthma in a randomized design by collecting three EBC samples within 7 days of the first.

### Pulmonary function

Spirometry was performed by means of a 10 L bell spirometer (Biomedin, Padova, Italy) and the best of three maneuvers, expressed as a percentage of predicted values, was chosen.

### Exhaled breath condensate collection

Exhaled breath condensate was collected using a condensing chamber (Ecoscreen, Jaeger, Hoechberg, Germany) as described previously [11]. Briefly, exhaled air entered and left the chamber through one-way valves at the inlet and outlet, thus keeping the chamber closed. Children were instructed to breath tidally through a mouthpiece connected to the condenser for 15 min. An average of 1.5 ml EBC per child was collected and stored a -80°C. LTB_4 _measurements were performed within 2 weeks from sample collection. α-Amylase concentrations in all EBC samples were measured by an in vitro colorimetric method to check for possible salivary contamination (Roche Diagnostics, Basel, Switzerland).

### Measurement of exhaled LTB_4_

Exhaled LTB_4 _was measured with a Finnigan Surveyor^®^LC System pump coupled with a TSQ Quantum Ultra™ triple quadrupole mass spectrometer using electrospray ionization with negative ion polarity mode with electrospray source (Thermo Electron Corporation, San José, USA). The LC/MS/MS experimental conditions were similar to those described previously [18]. However, the use of a Finnigan Surveyor^®^LC System pump coupled with a TSQ Quantum Ultra™ triple quadrupole mass spectrometer made it possible to increase 10 fold the analytical sensitivity (10 pg/ml vs 100 pg/ml). The lower limit of quantification (LOQ) was 50 pg/ml. The optimized source parameters were as follows: spray voltage, 4500 V; sheath gas pressure, 40 arbitrary units; aux gas pressure, 10 arbitrary units; capillary temperature, 300°C; capillary offset, 30 V; tube lens offset, 110 V. Chromatography was performed on a BetaBasic C18 column (15 cm × 2, 1 mm internal diameter, Thermo Hypersil-Keyston, Bellefonte, PA, USA) using a linear gradient with acetonitrile-water-acetic acid (30:70:0.05, v/v, at pH 5–6) which was changed to 100% acetonitrile over 4 minutes. Flow rate was 0.25 ml/min. LTB_4_-d_4 _(Cayman Chemicals Co., Ann Arbor, MI) was used as internal standard and was added to each sample to reach a final concentration of 1000 pg/ml. The peak area ratios for LTB_4_/LTB_4_-d_4 _were plotted versus LTB_4 _concentrations. The calibration curve in the range 10–500 pg/ml had a good linearity with r^2 ^= 0.9965. 10 μl of sample were directly injected into the liquid chromatograph without any pretreatment.

All solvents were high performance liquid chromatography grade obtained from Merck (Darmstadt, Germany); acetic acid extra pure was purchased by Riedel-de Haen, Sigma-Aldrich, Seelze, Germany. LTB_4 _and LTB_4_-d_4 _were purchased from Cayman Chemicals Co (Ann Arbor, MI, USA) and stored at -20°C until use. The working solutions were prepared daily using mobile phase as solvent.

LTB_4 _values in EBC were expressed as total amount (in picograms) of leukotriene expired in the 15-minute breath test (LT concentrations × volume of EBC). Concentration of LTB_4 _in undetectable samples was considered 25 pg/ml corresponding to 50% of the lower limit of quantification (50 pg/ml).

### Exhaled nitric oxide measurement

Exhaled NO was measured with the NIOX system (Aerocrine, Stockholm, Sweden) with a single breath on-line method according to American Thoracic Society guidelines [7]. Children inhaled NO-free air and exhaled through a dynamic flow restrictor. Children were asked to exhale to residual volume, insert the mouthpiece, inhale to total lung capacity through a NO filter, and then to exhale into the device through the same mouthpiece for 10 seconds at constant flow of 50 ml/sec with the aid of a visual feedback on a computer screen. A negative pressure tracing on the screen was used to confirm that children were inhaling NO-free air. Exhalations were repeated after 1-minute relaxation period until the performance of three exhaled NO values varied less than 10%. Exhaled NO measurements were obtained before spirometry.

### Statistical analysis

Linear regression analysis was used to assess the relationship between LC peak area ratios for LTB_4_/LTB_4_-d_4 _and LTB_4 _concentrations. Exhaled LTB_4 _and NO values were expressed as medians throughout with interquartile ranges (25^th ^to 75^th ^percentiles) shown in parentheses. Kruskal-Wallis test followed by pairwise Mann-Whitney U tests were used to compare groups. Correlations between variables were evaluated by Spearman test. Significance was defined as a value of p < 0.05.

## Results

No α-amylase concentrations (<22 mU/ml) were detected in any study sample, excluding significant salivary contamination.

### LTB_4 _in EBC

**Figure 1:**
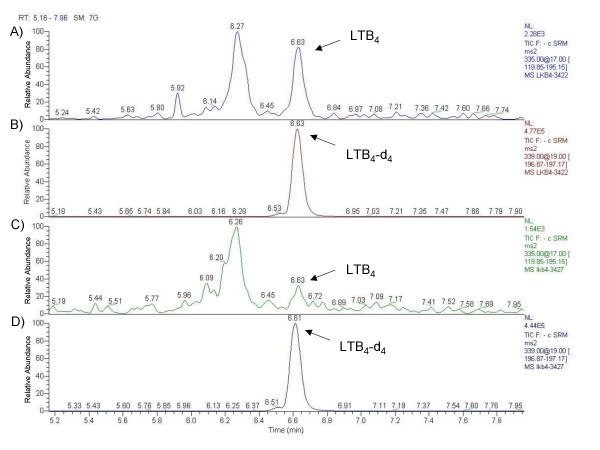
**Liquid chromatography/mass spectrometry-mass spectrometry chromatograms of exhaled breath condensate (EBC)**. Samples from an asthmatic child (A and B) and a healthy child (C and D) are shown. The ions m/z 195 (A and C) and m/z 197 (B and D) were used to monitor endogenous leukotriene (LT) B_4 _and LTB_4_-d_4 _(internal standard), respectively. LTB_4 _concentrations in EBC, calculated using peak area ratios for LTB_4_/LTB_4_-d_4_, were 87 pg/15 min (healthy child) and 450 pg/15 min (asthmatic child).

**Figure 2:**
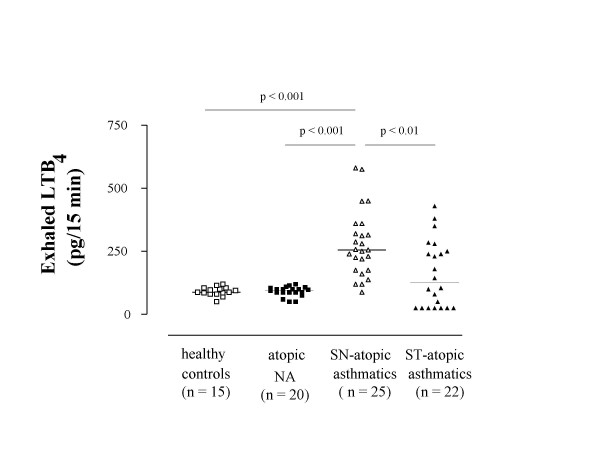
**Leukotriene (LT) B_4 _concentrations in exhaled breath condensate in children with allergic disease**. Exhaled LTB_4 _concentrations in healthy children (open squares), atopic non-asthmatic children (NA) (filled squares), steroid-naïve atopic asthmatic children (open triangles), and steroid-treated (ST) children with atopic asthma (filled triangles). LTB_4 _values are expressed as picograms produced during 15 minutes of breathing. Median values are shown with horizontal bars.

The mass spectra of LTB_4 _and LTB_4_-d_4 _revealed a base peak at *m/z *335 for endogenous LTB_4 _and *m/z *339 for internal standard LTB_4_-d_4_, corresponding to molecular ions. To increase specificity, LTB_4 _was detected in MS/MS mode. Parent ions *m/z *335 and *m/z *339 were fragmented with a collision energy of 17% and 19%, respectively. The transition 335 → 195 *m/z *and 339 → 197 *m/z *for internal standard was monitored (Figure 1). Exhaled LTB_4 _concentrations were detected in all steroid-naïve atopic asthmatics, atopic nonasthmatics, and healthy children and were undetectable in 7 steroid-treated atopic asthmatic children (Figure 2). Compared with healthy children [87.5 (82.5–102.5) pg], exhaled LTB_4 _was increased in steroid-naïve atopic asthmatic children [255.1 (175.0–314.7) pg, p < 0.001], but not in atopic nonasthmatic children [96.5 (87.3–102.5) pg, p = 0.59) (Figure 2). Steroid-naïve children with atopic asthma had higher exhaled LTB_4 _concentrations than atopic nonasthmatic children (p < 0.001) and healthy children (p < 0.001) (Figure 2).

**Figure 3:**
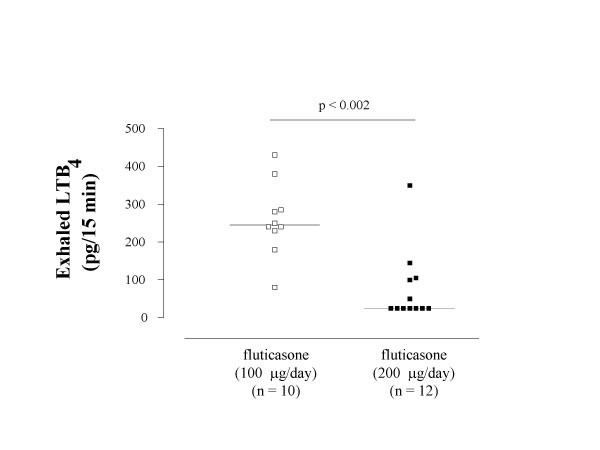
**Leukotriene (LT) B_4 _concentrations in exhaled breath condensate in steroid-treated children with atopic asthma**. Exhaled LTB_4 _concentrations in atopic asthmatic children who were receiving either 100 μg/day (open squares) or 200 μg/day (filled squares) of inhaled fluticasone at a constant dose for at least 8 weeks. LTB_4 _values are expressed as picograms produced during 15 minutes of breathing. Median values are shown with horizontal bars.

Asthmatic children who were receiving inhaled corticosteroids had lower concentrations of exhaled LTB_4 _than steroid-naïve asthmatics [125.0 (25.0–245.0) pg vs 255.1 (175.0–314.7) pg, p < 0.01, respectively] and similar to those in atopic nonasthmatic children (p = 0.41) and healthy controls (p = 0.43) (Figure 2). In steroid-treated atopic asthmatic children, subgroup analysis shows that children who were receiving 100 μg/day of inhaled fluticasone at a constant dose for at least 8 weeks had higher exhaled LTB_4 _values than those who were receiving with 200 μg/day of inhaled fluticasone at a constant dose for at least 8 weeks [245.0 (235.0–282.5) pg vs 25.0 (25.0–102.5) pg, p < 0.002, respectively) (Figure 3). In children receiving 200 μg/day of fluticasone, median exhaled LTB_4 _values were similar to those in atopic nonasthmatic children (p = 0.15) and healthy controls (p = 0.10). In contrast, exhaled LTB_4 _was elevated in asthmatic children who were receiving 100 μg/day of inhaled fluticasone (atopic nonasthmatic children: p < 0.005; healthy children: p < 0.001).

Seven asthmatic children who were treated with the higher dose of fluticasone had undetectable levels of exhaled LTB_4 _(Figure 3). MS/MS spectra of steroid-naïve asthmatic children (Figure 1A), healthy children (Figure 1C), and atopic nonasthmatic children (not shown) showed a major unknown peak in all samples. This peak is not always present in steroid-treated asthmatic children (not shown) and deserves further characterization. There was no correlation between exhaled LTB_4 _and exhaled NO in any study group. There was no correlation between exhaled LTB_4 _or exhaled NO and age, sex, or lung function in any study group. The intraclass correlation coefficient for LTB_4 _was 0.89.

### Exhaled NO

**Figure 4:**
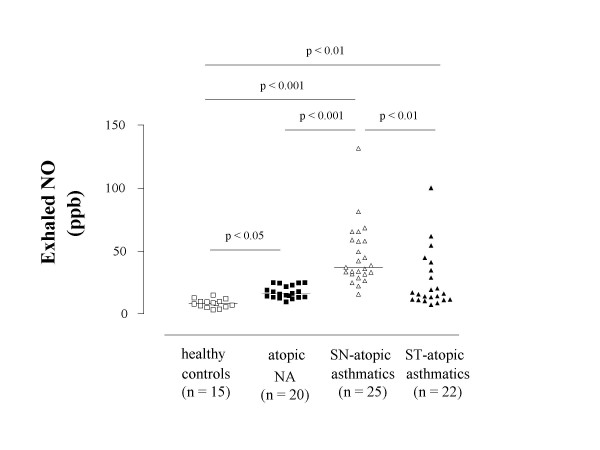
**Exhaled nitric oxide (NO) concentrations in children with allergic disease**. Exhaled NO concentrations in healthy children (open squares), atopic non-asthmatic children (NA) (filled squares), steroid-naïve atopic asthmatic children (open triangles), and steroid-treated (ST) children with atopic asthma (filled triangles). Median values are shown with horizontal bars.

**Figure 5:**
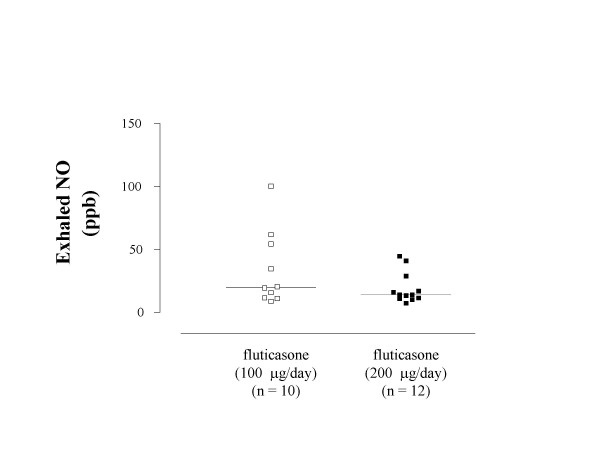
**Exhaled nitric oxide (NO) concentrations in exhaled breath condensate in steroid-treated children with atopic asthma**. Exhaled NO concentrations in atopic asthmatic children who were receiving either 100 μg/day (open squares) or 200 μg/day (filled squares) of inhaled fluticasone at a constant dose for at least 8 weeks. Median values are shown with horizontal bars.

Exhaled NO was higher in atopic nonasthmatic children [16.2 (13.5–22.4) ppb, p < 0.05] and, to a greater extent, in steroid-naïve atopic asthmatic children [37.0 (31.7–57.6) ppb, p < 0.001] than in healthy children [8.3 (6.1–9.9) ppb] (Figure 4). Compared with steroid-naïve asthmatic children, exhaled NO levels were reduced in asthmatic children who were receiving inhaled corticosteroids [15.9 (11.5–31.7) ppb, p < 0.01] (Figure 4). In these children, exhaled NO concentrations were higher than those in healthy controls (p < 0.01), but not than those in atopic nonasthmatic children (p = 0.98) (Figure 4). There was no difference in exhaled NO levels between asthmatic children receiving 200 μg/day of inhaled fluticasone and children receiving a daily dose of 100 μg [14.1 (11.3–22.9) ppb vs 19.8 (13.5–44.5) ppb, p = 0.27, respectively)] (Figure 5). Both subgroups of steroid-treated asthmatic children had higher exhaled NO levels than healthy children (children treated with fluticasone at a dose of 100 μg/day: p < 0.001; children treated with fluticasone at a dose of 200 μg/day: p < 0.001) but not than atopic nonasthmatic children (children treated with fluticasone at dose of 100 μg/day: p = 0.42; children treated with fluticasone at a dose of 200 μg/day: p = 0.46).

## Discussion

EBC is a completely non-invasive method for sampling secretions from the airways which is suitable for repeated measures of lung inflammation even in young children [19]. Using LTB_4_-d_4 _as internal standard, we have definitively demonstrated that LTB_4 _is present in the EBC, providing an accurate quantitative assessment of its concentrations in this biological fluid. In our study, absolute LTB_4 _values in EBC are significantly higher than those previously reported in studies using enzyme immunoassays [14,15], but similar to those found in studies in which exhaled LTB_4 _was measured by MS analysis [18,20]. The reasons for this discrepancy are unknown but could be partially related to matrix effects as differences in protein composition of the immunoassay buffer used for LTB_4 _standard dilution versus EBC samples might influence LTB_4 _measurement. We avoided possible matrix effects as EBC samples were directly injected into the chromatograph without any pre-treatment. Our study population of atopic asthmatic children had well maintained lung function as indicated by FEV_1 _values greater than 80% of predicted values, but evidence for small airway obstruction given that values of FEV_1_/FVC ratio and FEF_25–75%_, which are more sensitive for detecting reduced lung function in children [1], were decreased. Compared with healthy control children, exhaled LTB_4 _concentrations were selectively elevated in steroid-naïve children with atopic asthma, but not in atopic nonasthmatic children and in steroid-treated atopic asthmatic children. Enhanced airway inflammation in steroid-naïve children with asthma was confirmed by elevated exhaled NO levels in these children. These findings indicate that exhaled LTB_4 _can be used as a new in vivo non-invasive marker of inflammation in steroid-naïve asthmatic children.

We did not study the effects of allergen challenge on exhaled LTB_4 _in atopic nonasthmatic children and atopic asthmatic children. Similar exhaled LTB_4 _values in healthy children and atopic nonasthmatic children who had either perennial rhinitis or were recently exposed to a relevant allergen indicate that allergen exposure has little, if any, effect on exhaled LTB_4 _in atopic nonasthmatic children. However, allergen challenge studies to formally address this issue are warranted. The effect of allergen challenge on exhaled LTB_4 _levels in atopic asthmatic children is unknown and needs to be clarified.

In contrast to exhaled LTB_4_, exhaled NO levels were elevated in atopic nonasthmatic children although this increase was less pronounced than in steroid-naïve atopic asthmatic children. These findings and the lack of correlation between exhaled LTB_4 _and exhaled NO levels suggest a different biological significance for these two biomarkers as exhaled LTB_4 _might be considered as a marker of asthma, whereas exhaled NO might reflect the different degree of inflammation within the respiratory tract in atopic children with and without asthma. This may have important implications for diagnosis and treatment of children with allergic disease.

Measurement of exhaled LTB_4 _might help identify those children with no current respiratory symptoms, well-maintained lung function and elevated exhaled NO who are not only atopic but have ongoing asthmatic inflammation which may require earlier pharmacological therapy. Moreover, elevated exhaled NO levels in atopic nonasthmatic children with rhinitis reflect the presence of inflammation within the airways as the restricted exhalation method ensures velum closure excluding contamination of the exhaled air with nasal NO [7]. This is consistent with a common origin for allergic rhinitis and asthma [1]. However, larger studies are needed to assess the biological significance of these findings and their implications.

In the present study, median exhaled LTB_4 _concentrations in children who were receiving inhaled fluticasone were lower than those in steroid-naïve asthmatic children. A significant reduction in exhaled LTB_4 _levels was observed only in the group of children who were receiving 200 μg/day of inhaled fluticasone, whereas in children who were receiving a daily dose of inhaled fluticasone of 100 μg median exhaled LTB_4 _levels were similar to those in steroid-naïve asthmatic children. These findings might indicate that the lower dose of fluticasone is unable to reduce exhaled LTB_4 _levels in asthmatic children. However, there was a high individual variability in exhaled LTB_4 _concentrations in both subgroups of steroid-treated asthmatic children as well as in steroid-naïve asthmatic children which needs to be addressed in future studies. In a previous open-label uncontrolled study, treatment with inhaled fluticasone (100 μg twice a day for 4 weeks) had no effect on exhaled LTB_4 _concentrations in atopic asthmatic children with similar features [14]. This might be due to differences in the study design (open-label vs observational), duration of treatment (4 weeks vs at least 8 weeks), and the analytical technique used for measuring LTB_4 _in EBC (enzyme immunoassay vs LC/MS/MS) as concentrations of exhaled LTB_4 _measured by MS are 10-fold higher than those measured by immunoassay. However, the cross-sectional study design of the present study precludes definitive conclusions on the effect of inhaled corticosteroids on exhaled LTB_4 _in asthmatic children for which large controlled studies are required.

One limitation of the EBC analysis is that the cellular source of the inflammatory mediators within the airways cannot be ascertained. To this aim, invasive techniques such as bronchial biopsies are required.

To adjust for possible variations in the EBC sample volume, LTB_4 _values in EBC were expressed as total amount of the eicosanoid expired over a standard period of collection. However, as pointed out by Effros and coworkers, part of the variation in non-volatile compound concentrations in EBC may be related to differences in the dilution of respiratory droplets by water vapor [21]. The lack of correlation between structurally related compounds such as LTB_4 _and LTE_4 _reported in previous studies [13,14] does not seem to support this evidence. However, reference indicators such as measurement of conductivity as proposed by Effros and coworkers [22] should be used in future studies aiming at quantifying exhaled eicosanoids.

## Conclusion

Exhaled LTB_4 _is elevated in steroid-naïve atopic asthmatic children, but not in children receiving inhaled corticosteroids and in atopic nonasthmatic children. LC/MS/MS analysis of exhaled LTB_4 _might provide a completely non-invasive, sensitive, and quantitative method for airway inflammation assessment in asthmatic children. This technique is potentially applicable to patients with other lung diseases and suitable for longitudinal studies and assessing pharmacological therapy. The high cost is currently an important limitation. Further research is warranted for the complete characterization of the exhaled substances including cysteinyl-leukotrienes and for establishing the effect anti-asthmatic drugs on exhaled LTB_4_.

## Competing interests

The author(s) declare that they have no competing interests.

## Authors' contributions

PM conceived the idea for the study, collected exhaled breath condensate samples and measured exhaled nitric oxide. PM and PJB planned the investigation. CM recruited children and did clinical investigation and lung function tests. SM, MF and MC performed LC/MS analysis of leukotriene B_4 _in exhaled breath condensate. PM, PJB and MC participated in analysis and interpretation of data. PM wrote the report which was revised by PJB and MC. All authors read and approved the final manuscript.
